# Cerebrovascular events and outcomes in hospitalized patients with COVID-19: The SVIN COVID-19 Multinational Registry

**DOI:** 10.1177/1747493020959216

**Published:** 2020-09-30

**Authors:** James E Siegler, Pere Cardona, Juan F Arenillas, Blanca Talavera, Ana N Guillen, Alba Chavarría-Miranda, Mercedes de Lera, Priyank Khandelwal, Ivo Bach, Pratit Patel, Amit Singla, Manuel Requena, Marc Ribo, Dinesh V Jillella, Srikant Rangaraju, Raul G Nogueira, Diogo C Haussen, Alejandro R Vazquez, Xabier Urra, Ángel Chamorro, Luis S Román, Jesse M Thon, Ryna Then, Emma Sanborn, Natalia P de la Ossa, Mònica Millàn, Isaac N Ruiz, Ossama Y Mansour, Mohammed Megahed, Cristina Tiu, Elena O Terecoasa, Răzvan A Radu, Thanh N Nguyen, Gioacchino Curiale, Artem Kaliaev, Alexandra L Czap, Jacob Sebaugh, Alicia M Zha, David S Liebeskind, Santiago Ortega-Gutierrez, Mudassir Farooqui, Ameer E Hassan, Laurie Preston, Mary S Patterson, Saif Bushnaq, Osama Zaidat, Tudor G Jovin

**Affiliations:** 1 Cooper Neurologic Institute, Cooper University Hospital, Camden, NJ, USA; 2 Cooper Medical School of Rowan University, Camden NJ, USA; 3 Department of Neurology, Hospital Universitari, Bellvitge, Barcelona, Spain; 4 Department of Neurology, Hospital Clínico Universitario, Valladolid, Spain; 5 Neurovascular Research Laboratory, Instituto de Biología y Genética Molecular, Universidad de Valladolid, Consejo Superior de Investigaciones Científicas, Madrid, Spain; 6 Department of Neurology, Robert Wood Johnson University Hospital, New Brunswick, NJ, USA; 7 New Jersey Medical School, Newark, NJ, USA; 8 Department of Neurosurgery, Robert Wood Johnson University Hospital, New Brunswick, NJ, USA; 9 Stroke Unit, Department of Neurology, Vall d'Hebron Research Institute, Barcelona, Spain; 10 Departament de Medicina, Universitat Autónoma de Barcelona, Barcelona, Spain; 11 Department of Neurology, Emory University School of Medicine, Atlanta, GA, USA; 12 Department of Neurology, Grady Memorial Hospital, Atlanta, GA, USA; 13 Department of Neurology, Hospital Clínic, Barcelona, Spain; 14 Area of Neuroscience, Institut d’Investigacions Biomèdiques August Pi I Sunyer (IDIBAPS), Barcelona, Spain; 15 Department of Radiology, Hospital Clínic, Barcelona, Spain; 16 Stroke Unit, Neuroscience Department, Hospital Universitari Germans Trias i Pujol, Carretera Canyet s/n, Badalona, Barcelona, Spain; 17 Department of Neurology, Stroke and Neurointervention division, Alexandria University, Alexandria, Egypt; 18 Department of Critical Care Medicine, Alexandria University, Alexandria, Egypt; 19 Department of Neurology, University Emergency Hospital Bucharest, Bucharest, Romania; 20 “Carol Davila” University of Medicine and Pharmacy, Bucharest, Romania; 21 Department of Neurology, Boston Medical Center, Boston University School of Medicine, MA, USA; 22 Department of Radiology, Boston Medical Center, Boston University School of Medicine, MA, USA; 23 Department of Neurosurgery, Boston Medical Center, Boston University School of Medicine, MA, USA; 24 Department of Neurology, McGovern Medical School, University of Texas Health Science Center, Houston, TX, USA; 25 Department of Neurology, Ronald Reagan UCLA Medical Center, Los Angeles, CA, USA; 26 Department of Neurology, Neurosurgery and Radiology, University of Iowa Hospitals and Clinics, Iowa City, IA, USA; 27 Department of Clinical Neuroscience Research, Valley Baptist Medical Center, Harlingen, TX, USA; 28 Department of Neurology, University of Texas Rio Grande Valley, Harlingen, TX, USA; 29 Neuroscience Institute, Bon Secours Mercy Health St. Vincent Hospital, Toledo, OH, USA

**Keywords:** All cerebrovascular diseases/stroke, intracranial hemorrhage, cerebral venous thrombosis, COVID-19

## Abstract

**Background:**

Severe acute respiratory syndrome-coronavirus-2 (SARS-CoV-2) has been associated with a significant risk of thrombotic events in critically ill patients.

**Aim:**

To summarize the findings of a multinational observational cohort of patients with SARS-CoV-2 and cerebrovascular disease.

**Methods:**

Retrospective observational cohort of consecutive adults evaluated in the emergency department and/or admitted with coronavirus disease 2019 (COVID-19) across 31 hospitals in four countries (1 February 2020–16 June 2020). The primary outcome was the incidence rate of cerebrovascular events, inclusive of acute ischemic stroke, intracranial hemorrhages (ICH), and cortical vein and/or sinus thrombosis (CVST).

**Results:**

Of the 14,483 patients with laboratory-confirmed SARS-CoV-2, 172 were diagnosed with an acute cerebrovascular event (1.13% of cohort; 1130/100,000 patients, 95%CI 970–1320/100,000), 68/171 (40.5%) were female and 96/172 (55.8%) were between the ages 60 and 79 years. Of these, 156 had acute ischemic stroke (1.08%; 1080/100,000 95%CI 920–1260/100,000), 28 ICH (0.19%; 190/100,000 95%CI 130–280/100,000), and 3 with CVST (0.02%; 20/100,000, 95%CI 4–60/100,000). The in-hospital mortality rate for SARS-CoV-2-associated stroke was 38.1% and for ICH 58.3%. After adjusting for clustering by site and age, baseline stroke severity, and all predictors of in-hospital mortality found in univariate regression (p < 0.1: male sex, tobacco use, arrival by emergency medical services, lower platelet and lymphocyte counts, and intracranial occlusion), cryptogenic stroke mechanism (aOR 5.01, 95%CI 1.63–15.44, p < 0.01), older age (aOR 1.78, 95%CI 1.07–2.94, p = 0.03), and lower lymphocyte count on admission (aOR 0.58, 95%CI 0.34–0.98, p = 0.04) were the only independent predictors of mortality among patients with stroke and COVID-19.

**Conclusions:**

COVID-19 is associated with a small but significant risk of clinically relevant cerebrovascular events, particularly ischemic stroke. The mortality rate is high for COVID-19-associated cerebrovascular complications; therefore, aggressive monitoring and early intervention should be pursued to mitigate poor outcomes.

## Introduction

Thrombotic complications of the novel human coronavirus responsible for the systemic acute respiratory syndrome, SARS-CoV-2, have been well described.^1,2^ Early reports from Wuhan, China, indicated a 2–5% risk of acute ischemic stroke among hospitalized patients with the coronavirus disease 2019 (COVID-19).^3^ Other series have reported that patients with SARS-CoV-2 are at risk of embolic strokes with severe deficits and a high in-hospital mortality rate.^4–6^ This risk may be more than seven times greater for patients with SARS-CoV-2 than patients with influenza according to one comparative analysis.^7^ Although initial estimates indicated a 1 in 20 risk of stroke in patients with COVID-19, more recent data report a rate of 1–2% among hospitalized patients.^7^

## Aims

To better illustrate the relationship between SARS-CoV-2 and associated cerebrovascular events (CVE), we established a multinational registry to estimate the global incidence rate of these neurologic complications among consecutively evaluated patients with COVID-19. Furthermore, given the high short-term mortality of hospitalized patients with COVID-19 (5–20%),^8,9^ we sought to evaluate the short-term functional outcomes and survival of patients with COVID-19 and associated cerebrovascular disease.

## Methods

### Study design and participants

A retrospective observational registry involving prospectively gathered data from 17 healthcare networks (31 unique hospitals) across four countries (USA, Spain, Egypt, and Romania) was queried. Participating centers were recruited based on affiliation with the Society of Vascular and Interventional Neurology (SVIN) and contacts among site investigators. All consecutive patients ≥18 years of age who were evaluated in each center’s emergency department (ED) and/or admitted between 1 February 2020 and 16 June 2020 were eligible for inclusion. To be included, patients must have been diagnosed with SARS-CoV-2 using oropharyngeal polymerase chain reaction (PCR) or IgG and/or IgM antibody presence in the sera using commercial assays. All imaging to confirm the diagnosis of acute ischemic stroke, intracranial hemorrhage, or cortical vein and/or dural sinus thrombosis (CVST) was performed and interpreted at the discretion of the treating physician. This study was approved under a waiver of informed consent by the local institutional review board at each participating center, and it is reported in accordance with the STrengthening the Reporting of OBservational studies in Epidemiology statement.

### Data collection

Patient demographic information, including age, sex, race, and ethnicity, as well as pertinent medical history, National Institutes of Health Stroke Scale (NIHSS) score, neuroimaging, treatment, laboratory testing (hematologic parameters, d-dimer), functional outcome at the time of discharge using the modified Rankin Scale (mRS),^10,11^ and discharge disposition were captured. De-identified data elements (including age, which was binned by decade for de-identification purposes) were documented by local investigators on a HIPAA-compliant, online platform, as previously described,^12^ and were made accessible to the data coordinating center (Cooper University Hospital) for analysis. Intracranial occlusion was defined broadly as any intracranial arterial occlusion identified on vascular imaging. Stroke etiology was classified according to the Trial of Org 10172 in Acute Stroke Treatment as previously described.^13^ Sites also reported their overall in-hospital mortality rate among all COVID-19 patients throughout the study period. Data elements with a <50% completion rate (e.g. intubation and/or tracheostomy, length of hospital stay) were not reported in order to limit the selection bias. Ninety-day outcomes (including mRS, living situation, and survival) were unavailable given the recency of the study period and the generally protracted hospitalizations for COVID-19 patients.

### Statistical analysis

No sample-size calculations were performed prior to data collection. Descriptive statistics were used to summarize continuous and categorical variables. Normality of continuous data was assessed histographically and confirmed using the Shapiro–Wilk test. Continuous variables were reported as medians with interquartile range. The primary outcome was the incidence rate of CVEs, which was summated by healthcare institutions and across the entire cohort, with approximate binomial 95% confidence intervals (95%CI) per 100,000 hospitalized COVID-19 patients using the Agresti–Coull method.^14^ After event rates were found to vary according to site COVID-19 volume, Pearson’s correlation coefficient was calculated to estimate the association between the COVID-19 volume at individual sites and (1) CVE incidence rate, (2) stroke incidence rate, and (3) in-hospital mortality.

As part of the secondary objective, we evaluated the clinical, laboratory, and radiographic factors that were associated with in-hospital mortality. In addition to using the aforementioned descriptive statistics, the two-sample test of proportions was used to determine significant differences in-hospital mortality between each of the various stroke mechanisms. All relevant covariates were entered into univariate regression models estimating the odds of mortality among patients with ischemic stroke. Variables significant to p < 0.1 in these models were added to age and baseline NIHSS (known significant predictors of mortality)^15,16^ in a multivariable logistic regression model, which was clustered by site. A separate multivariable model was generated among patients with ischemic stroke due to an intracranial occlusion estimating the odds of in-hospital mortality, incorporating age, baseline NIHSS, and all variables significant to p < 0.1 in univariate regression. All tests were performed at the two-sided level using STATA 15.0 (College Station, TX), with p ≤ 0.05 considered statistically significant. As this was an exploratory analysis, p-values are reported for convention and should be interpreted with caution. No adjustments were made for multiple comparisons. Missing data were not imputed. Fully de-identified data will be made available upon reasonable request of the corresponding author.

## Results

**Figure 1.**
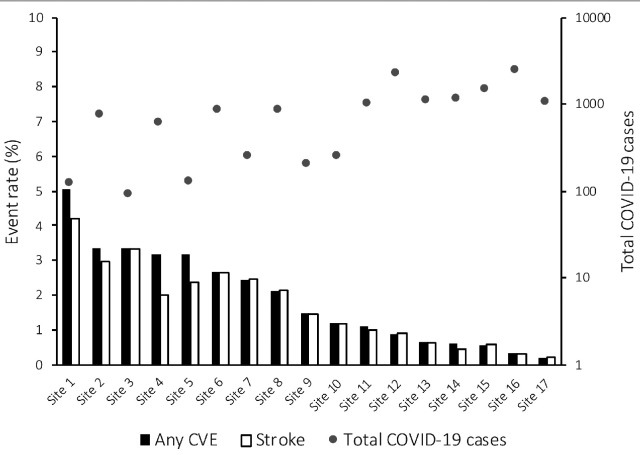
Prevalence of CVEs by site. Incidence rate of any CVEs and stroke are plotted using vertical bars (primary y-axis) while the total COVID-19 ED and/or hospitalization rate are plotted as dots (secondary y-axis). CVE: cerebrovascular event; COVID-19: coronavirus disease 2019.

Of the 14,483 patients with laboratory-confirmed SARS-CoV-2, 172 were diagnosed with an acute CVE (1.13% of COVID-19 cohort; 1130 per 100,000 patients, 95%CI 970–1320 per 100,000). Among patients with CVE, 68/171 (40.5%) were female, 94/138 (68.1%) with reported race were White, and 96/172 (55.8%) were between the ages 60 and 79 years. Between sites, CVE rates ranged from 0.19 to 5.04% (Figure 1), with sites treating more COVID-19 patients reporting a lower rate of CVE (r =−0.659, p = 0.004) and acute ischemic stroke (r = −0.668, p = 0.003). The most common CVE was acute ischemic stroke, observed in 156 patients (1.08%; 1080 per 100,000, 95%CI 920–1260 per 100,000), with 28 patients having radiographically confirmed primary ICH (0.19%; 190 per 100,000, 95%CI 130–280 per 100,00) and 3 with CVST (0.02%; 20 per 100,000, 95%CI 4–60 per 100,000). Fourteen patients had acute stroke and intracerebral hemorrhage (8.1% of cohort), with a similar distribution of age and comorbidities as the overall stroke population in this cohort (Table 1).

**Table 1.** Demographic data

	All patients with suspected or radiographically confirmed CVE^a^ (*n* = 172)	Acute ischemic stroke (*n* = 156)	Intracranial hemorrhage (*n* = 28)	Stroke + intracranial hemorrhage (*n* = 14)
Age, no. (%)				
<30	0/172 (0%)	0/156 (0%)	0/28 (0%)	0/14 (0%)
30–39	5/172 (2.9%)	4/156 (2.6%)	1/28 (3.6%)	0/14 (0%)
40–49	19/172 (11.1%)	15/156 (9.6%)	6/28 (21.4%)	3/14 (21.4%)
50–59	24/172 (14.0%)	20/156 (12.8%)	4/28 (14.3%)	0/14 (0%)
60–69	50/172 (29.1%)	49/156 (31.4%)	4/28 (14.3%)	4/14 (28.6%)
70–79	46/172 (26.7%)	45/156 (28.9%)	5/28 (17.8%)	4/14 (28.6%)
80–89	23/172 (13.4%)	18/156 (11.5%)	8/28 (28.9%)	3/14 (21.4%)
>89	5/172 (2.9%)	5/156 (3.2%)	0/28 (0%)	0/14 (0%)
Sex, no. female (%)	68/171 (40.5%)	61/155 (39.4%)	10/28 (35.7%)	4/14 (28.6%)
Race, no. (%)				
White	94/138 (68.1%)	84/125 (67.2%)	20/24 (83.3%)	10/12 (83.3%)
Black	37/138 (26.8%)	30/125 (28.0%)	3/24 (12.5%)	2/12 (16.7%)
Asian	3/138 (2.2%)	2/125 (1.6%)	1/24 (4.2%)	0/12 (0%)
More than one race	2/138 (1.5%)	2/125 (1.6%)	0/24 (0%)	0/12 (0%)
Other	2/138 (1.5%)	2/125 (1.6%)	0/24 (0%)	0/12 (0%)
Hispanic ethnicity, no. (%)	62/154 (40.3%)	58/140 (41.4%)	10/27 (37.0%)	6/14 (42.9%)
Method of arrival, no. (%)				
Emergency medical services	67/115 (58.3%)	59/105 (56.2%)	12/19 (63.2%)	5/10 (50.0%)
Transfer from outside hospital	30/115 (26.1%)	28/105 (26.7%)	6/19 (31.6%)	4/10 (40.0%)
Walk-in/private vehicle	13/115 (11.3%)	13/105 (12.4%)	1/19 (5.3%)	1/10 (10.0%)
Other	5/113 (4.4%)	5/105 (4.8%)	0/19 (0%)	0/10 (0%)
Diagnosis of COVID-19,^b^ no. (%)				
Oropharyngeal PCR	169/172 (98.3%)	153/156 (98.1%)	26/28 (92.9%)	12/14 (85.7%)
Serum IgM and/or IgG	12/172 (1.7%)	11/156 (7.1%)	2/28 (7.1%)	2/14 (14.3%)
Known COVID-19 exposure, no. (%)	22/172 (12.8%)	19/156 (12.2%)	3/28 (10.7%)	0/14 (0%)
Medical history, no. (%)				
Hypertension	121/170 (71.2%)	111/154 (72.1%)	18/28 (64.3%)	9/14 (64.3%)
Diabetes mellitus	68/168 (40.5%)	65/152 (42.8%)	7/28 (25.0%)	5/14 (35.7%)
Dyslipidemia	61/155 (39.4%)	58/142 (40.1%)	9/25 (36.0%)	6/13 (46.2%)
Atrial fibrillation	30/163 (18.4%)	27/148 (18.2%)	5/22 (18.5%)	2/14 (14.3%)
Congestive heart failure	30/170 (17.7%)	27/154 (17.5%)	6/22 (21.4%)	4/10 (40.0%)
Active tobacco use	17/150 (10.8%)	15/145 (10.3%)	3/26 (11.5%)	1/14 (7.1%)
Prior stroke	19/154 (12.3%)	15/140 (10.7%)	6/27 (22.2%)	2/14 (14.3%)
Chronic renal insufficiency (stage III/IV or dialysis-dependent)	18/160 (11.3%)	16/144 (11.1%)	5/28 (17.9%)	3/14 (21.4%)
Chronic obstructive pulmonary disease and/or asthma	12/148 (8.1%)	11/135 (8.2%)	2/26 (7.7%)	1/14 (7.1%)
Cancer	9/147 (6.1%)	8/134 (6.0%)	4/26 (15.4%)	3/14 (21.4%)

a CVE includes acute ischemic stroke, intracranial hemorrhage, or cortical vein and/or dural sinus thrombosis.

b 9 patients with a CVE, including 8 patients with an acute stroke were diagnosed with COVID-19 using both serum antibodies and oropharyngeal PCR.

CVE: cerebrovascular event; COVID-19: coronavirus disease 2019; PCR: polymerase chain reaction.

Demographic data are summarized in Table 1. Deficits were generally moderate or severe among patients with stroke (median NIHSS 13 (IQR 5–21)) or ICH (median NIHSS 22 (IQR 7–25)). The most common mechanism of acute stroke among COVID-19 patients was cryptogenic (42.6%) followed by cardioembolism (27.1%). An intracranial occlusion was identified in 53/107 patients with intracranial arterial imaging (49.5%; Table 2).

**Table 2.** Clinical and radiographic features

	Acute ischemic stroke (*n* = 156)	Intracranial hemorrhage (*n* = 28)
NIHSS at baseline, median (IQR)	13 (5–21) (*n* = 130)	22 (7–25) (*n* = 16)
Glasgow Coma Scale at baseline, median (IQR)	n/a	6 (4–15) (*n* = 15)
Head CT performed, no. (%)	135/151 (89.4%)	28 (100%)
First CT indicating acute stroke, no. (%)	76/132 (57.6%)	n/a
Intracranial hemorrhage on CT, no. (%)	14/132 (10.6%)	28 (100%)
Any vascular imaging performed, no. (%)	105/156 (67.3%)	14/28 (50.0%)
Intracranial occlusion identified, no. (%)	53/107 (49.5%)	n/a
MRI brain performed, no. (%)	55/151 (36.4%)	8/27 (29.6%)
MRI evidence of acute stroke, no. (%)	45/55 (81.8%)	5/8 (62.5%)
CT or MRI location of infarction, no. (%)		n/a
Cortical	81/101 (80.2%)	n/a
Subcortical supratentorial	54/101 (53.5%)	n/a
Infratentorial	12/101 (11.9%)	n/a
Stroke etiology, no. (%)		
Cardioembolism	35/129 (27.1%)	n/a
Large vessel atherosclerotic disease	15/129 (11.6%)	n/a
Small vessel disease	4/129 (3.1%)	n/a
Cryptogenic	55/129 (42.6%)	n/a
Other	11/129 (8.5%)	n/a
ICH compartment,^a^ no. (%)		
Intraparenchymal	n/a	20 (71.4%)
Subarachnoid	n/a	7 (25.0%)
Subdural	n/a	2 (7.1%)
Intraventricular	n/a	8 (28.6%)
Epidural	n/a	0 (0%)
IPH location*, no. (%)		
Supratentorial	n/a	13/18 (72.2%)
Infratentorial	n/a	7/18 (38.9%)
IPH volume, median cc (IQR)	n/a	20 (10–66.2) (n = 11)
ICH score, median (IQR)	n/a	3 (1–3) (n = 10)
IPH etiology, no. (%)		
Coagulopathy	n/a	8/16 (50.0%)
Hypertension	n/a	5/16 (31.3%)
Other	n/a	3/16 (18.8%)

a Some patients experienced intracranial hemorrhage in multiple compartments with intraparenchymal involvement of supra- and infratentorial regions.

NIHSS: National Institutes of Health Stroke Scale; IQR: interquartile range; CT: computed tomography; MRI: magnetic resonance imaging; ICH: intracranial hemorrhage; IPH: intraparenchymal hemorrhage.

**Figure 2.**
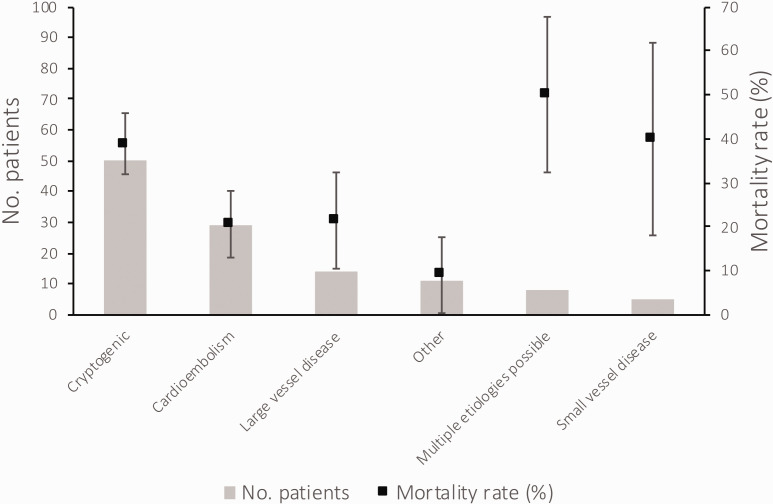
In-hospital mortality rate according to stroke etiology. The distribution of stroke etiologies is plotted using vertical bars (primary y-axis), and mortality rate according to stroke mechanism is plotted as squares with standard error bars (secondary y-axis).

The median in-hospital COVID-19 mortality rate was 11.7% (IQR 8.6–18.4%), with no correlation between COVID-19 volume and in-hospital mortality among COVID-19 patients (r = 0.002), or among patients with COVID-19 and CVE (r = 0.01; Supplemental Figure 1). There was a non-significant variation in mortality across sites (p = 0.09), but this did not vary significantly by site COVID-19 volume (Supplemental Figures 1 and 2). More than one-third of patients with acute stroke and COVID-19 died during hospitalization, while more than half of patients with ICH and COVID-19 died (Supplemental Table 1). The median time from stroke onset to death was four days (IQR 1–10) among patients with stroke, 33 of whom died within seven days (21.2% of all stroke patients). The median time from ICH onset to death was 3.5 days (IQR 1–7), 11 of whom died within seven days (39.3% of all ICH patients). Although there was no significant difference in mortality according to stroke mechanism (p = 0.22), there was a significantly higher mortality rate among patients with stroke plus intracranial hemorrhage (OR 4.07, 95%CI 1.19–13.85, p = 0.03) and a non-significantly higher mortality for stroke of cryptogenic mechanism versus stroke due to “other” mechanisms (p = 0.07), and cryptogenic strokes versus strokes due to cardioembolism (p = 0.098; Figure 2). Among all patients with a stroke, those more likely to die were male (OR 2.16, 95%CI 1.04–4.48, p = 0.04), had more severe deficits (β = 0.017, 95%CI 0.009–0.026, p < 0.001), and a cryptogenic stroke mechanism (OR 2.08, 95%CI 0.94–4.63, p = 0.07). When added to age (p = 0.03 in unadjusted regression) in the final multivariable model after adjustment for all variables significant to p < 0.1 in univariate regression (male sex, tobacco use, arrival by emergency medical services, lower admission platelet and lymphocyte counts, and intracranial occlusion)—and after clustering by site—cryptogenic stroke mechanism (aOR 5.01, 95%CI 1.63–15.44, p < 0.01), older age (aOR 1.78, 95%CI 1.07–2.94, p = 0.03), and lower lymphocyte count on admission (aOR 0.58, 95%CI 0.34–0.98 p = 0.04) remained the only independent predictors of in-hospital mortality (Supplemental Table 2). Cryptogenic stroke also remained the only independent predictor of in-hospital mortality among patients with an intracranial occlusion (aOR 5.03, 95%CI 1.99–12.74, p < 0.01; Supplemental Table 3).

## Discussion

In this observational cohort of more than 14,000 COVID-19 patients treated at 31 hospitals in four countries, approximately 1 in 100 patients were diagnosed with a clinically relevant or radiographically confirmed cerebral infarction. The reported incidence rate of any CVE varied between 0.19 and over 5% in this cohort, with a more than twofold higher rate of cryptogenic ischemic stroke (42%) when compared to historic cohorts (∼17%)^17^ and half of patients having an embolic occlusion. The high prevalence of cryptogenic infarctions with an embolic radiographic appearance validates prior reports suggesting a prothrombotic mechanism of stroke among patients with COVID-19.^7^ Furthermore, the severity of disease in patients with a cryptogenic (SARS-CoV-2-associated) stroke is reflected by the high mortality rate among cryptogenic stroke patients with COVID-19 (39%). Importantly, the early mortality risk resonates with other reports of increased mortality with SARS-CoV-2 among patients with comorbid cerebrovascular disease,^18^ although it vastly exceeds what has been historically described in non-SARS-CoV2 patients (6% at one year for patients with cryptogenic stroke vs. 21% for cardioembolism and large vessel disease).^19^ This suggests that COVID-19-associated cryptogenic stroke is a truly unique stroke mechanism with a high probability of early mortality. Whether stroke is a driving factor for early mortality in COVID-19, or a clinical biomarker of disease severity, should be explored in future studies.

An unrelated observation was that the hospital COVID-19 volume was strongly and inversely correlated with the reported CVE rates. Several explanations may account for this. First, sites with higher volumes of COVID-19 patients may have broader testing or admission criteria for asymptomatic or mild patients, among whom a cerebrovascular complication may be less common. Second, sites with higher volumes of COVID-19 patients may have experienced significant resource limitations and as such, may have missed stroke diagnoses among critically ill hospitalized patients with COVID-19. Third, sites responsible for rapid triage and management of a large number of COVID-19 patients may have sought to limit healthcare provider exposure to patients or restrict certain diagnostic testing (e.g. neuroimaging with CT and/or MRI) to prevent delays in the care of other patients while these resources could be decontaminated. And finally, sites with higher COVID-19 volumes may have implemented more aggressive antithrombotic treatment earlier in the course of hospitalization for COVID-19. However, data regarding prophylactic treatment was not captured in this investigation.

Our study also validates recent data from a New York health system that reported COVID-19 patients with acute stroke had a higher probability of cryptogenic embolic stroke mechanism with more severe deficits.^8^ Most patients in our cohort had moderate or severe deficits according to the NIHSS, with an 80% prevalence of cortical involvement on neuroimaging and 50% rate of intracranial occlusion—consistent with an embolic mechanism. It is worth noting that while strokes in this cohort were more severe than in historic cohorts, we did not account for critical illness and/or sedation, which could have artificially augmented the NIHSS in these patients. Regardless of confounding by sedation or critical illness, the observation that COVID-19 patients with cryptogenic strokes (presumably related to the prothrombotic milieu triggered by SARS-CoV-2) were significantly more likely to die during their hospitalization speaks to the gravity of cerebrovascular complications. More importantly, it should galvanize our efforts to closely monitor for such complications and manage aggressively when an event is suspected.

## Limitations

While this study is the largest observational cohort of consecutive COVID-19 patients with cerebrovascular complications to date, it is limited by its retrospective nature, inclusion of patients largely representing the United States and Spain, and unavailability of some outcome data (e.g. length of stay, 90-day functional status). Because we prioritized data collection for hospitalized patients with COVID-19 and CVE, we are unable to report any comparisons between patients with CVE and those without CVE. The incidence of CVE observed in this cohort is not reflective of the overall incidence of CVE among COVID-19 patients because these data were limited to sick patients who presented to the ED and/or were hospitalized. In addition, sites participating in this study were affiliated with the SVIN, with large referral networks, which could have confounded the severity of cases and prevalence of intracranial occlusion. However, among patients who arrived via transfer from outside institutions, there was no higher prevalence of an occlusion (6/36 vs. 10/35, p = 0.23) or greater severity of deficits (median NIHSS 17 vs. 11, p = 0.47) when compared to patients who arrived by other means (data not otherwise shown). In a separately reported analysis involving 11 of the sites included here, the rate of intracranial occlusion among all-comers with stroke admitted during a similar study period was 19.6% (183/933 patients, unpublished data), which is slightly lower than what has been reported in observational studies (∼30%).^20^ Therefore, we do not believe that this COVID-19 population reflects institutional biases which could lead to a higher rate of LVO patients, irrespective of COVID-19 status. While we observed a high rate of cryptogenic stroke in this COVID-19 population, the diagnosis of a cryptogenic mechanism (and minimum diagnostic testing for stroke evaluation—including long-term cardiac event monitoring, intracranial vascular imaging, etc.) was left to the discretion of the treating physician. It is possible that patients with cryptogenic stroke had less comprehensive diagnostic evaluations due to severity of their illness and low probability of survival, which could have confounded these results. However, the time from stroke onset to death was a median of four days, indicating that a timely evaluation could have been pursued to rule out traditional mechanisms of stroke. Stroke mechanisms in this COVID-19 population are being reported separately. Furthermore, these data reflect the experience of healthcare centers within and associated with the SVIN network, with 62% of patients being treated outside the United States. Importantly, the primary objective of this study was to estimate the incidence rate of CVE and outcomes at healthcare institutions that could monitor for neurologic complications of COVID-19. More than 80% of patients with acute ischemic stroke and 100% of patients with ICH and CVST had radiographically confirmed events. It is possible, if not probable, that the lack of serial neuroimaging of critically ill patients with COVID-19 may underestimate the event rates reported in this multicenter study. The question of how best to prevent thrombotic complications in patients with COVID-19 also remains unanswered. The non-randomized allocation of treatment in the form of antiplatelet therapy (single or dual), anticoagulation, or both, in conjunction with the small number of total stroke patients, precludes a meaningful comparison of treatment effects. The best course of treatment can only be clarified by randomized clinical trials evaluating immunotherapies and antithrombotics or by pooling of these data with other observational studies.

## Conclusions

The arterial thrombotic complications of COVID-19 are not to be understated. While the risk of a clinically relevant stroke was low in this multicenter study, there was considerable variability in the incidence rate (as high as 5%), and the risk of poor outcomes was significant. This builds on existing literature suggesting the stroke risk to be ∼1%;^7,8^ however, the risk may be higher in some populations or those with more frequent neuroimaging and/or aggressive neurologic monitoring. It is likely that this approximately 1% risk of clinically significant stroke reflects a floor rate, and the true rate could be even higher. In our cohort of geographically distinct and medically unique patients, the observed high mortality rate validates one recent meta-analysis of 4448 patients with COVID-19^21^ using a larger multicenter data set. Patients with stroke may have suffered from a more virulent strain of SARS-CoV-2^22^ and/or greater complications of COVID-19, which puts them at a higher risk of long-term disability and in-hospital mortality. The severity of cerebrovascular disease during the COVID-19 pandemic may be compounded by the lack of in-hospital family support with visitor restrictions, patients being managed in a COVID-19 unit rather than a dedicated stroke unit, and possibly less contact with physical therapy and occupational services until patients are liberated from COVID-19 precautions. However, it may also be that the thrombotic complications of the virus could drive the adverse outcomes. Additional studies are called upon to firmly establish COVID-19 as a unique mechanism of stroke and to better characterize the long-term complications of COVID-19-associated cerebrovascular disease.

## Supplemental Material

sj-jpg-1-wso-10.1177_1747493020959216 - Supplemental material for Cerebrovascular events and outcomes in hospitalized patients with COVID-19: The SVIN COVID-19 Multinational Registry Click here for additional data file. Supplemental material, sj-jpg-1-wso-10.1177_1747493020959216 for Cerebrovascular events and outcomes in hospitalized patients with COVID-19: The SVIN COVID-19 Multinational Registry by James E Siegler, Pere Cardona, Juan F Arenillas, Blanca Talavera, Ana N Guillen, Alba Chavarría-Miranda, Mercedes de Lera, Priyank Khandelwal, Ivo Bach, Pratit Patel, Amit Singla, Manuel Requena, Marc Ribo, Dinesh V Jillella, Srikant Rangaraju, Raul G Nogueira, Diogo C Haussen, Alejandro R Vazquez, Xabier Urra, Ángel Chamorro, Luis S Román, Jesse M Thon, Ryna Then, Emma Sanborn, Natalia P de la Ossa, Mònica Millàn, Isaac N Ruiz, Ossama Y Mansour, Mohammed Megahed, Cristina Tiu, Elena O Terecoasa, Răzvan A Radu, Thanh N Nguyen, Gioacchino Curiale, Artem Kaliaev, Alexandra L Czap, Jacob Sebaugh, Alicia M Zha, David S Liebeskind, Santiago Ortega-Gutierrez, Mudassir Farooqui, Ameer E Hassan, Laurie Preston, Mary S Patterson, Saif Bushnaq, Osama Zaidat and Tudor G Jovin in International Journal of Stroke

sj-jpg-2-wso-10.1177_1747493020959216 - Supplemental material for Cerebrovascular events and outcomes in hospitalized patients with COVID-19: The SVIN COVID-19 Multinational Registry Click here for additional data file. Supplemental material, sj-jpg-2-wso-10.1177_1747493020959216 for Cerebrovascular events and outcomes in hospitalized patients with COVID-19: The SVIN COVID-19 Multinational Registry by James E Siegler, Pere Cardona, Juan F Arenillas, Blanca Talavera, Ana N Guillen, Alba Chavarría-Miranda, Mercedes de Lera, Priyank Khandelwal, Ivo Bach, Pratit Patel, Amit Singla, Manuel Requena, Marc Ribo, Dinesh V Jillella, Srikant Rangaraju, Raul G Nogueira, Diogo C Haussen, Alejandro R Vazquez, Xabier Urra, Ángel Chamorro, Luis S Román, Jesse M Thon, Ryna Then, Emma Sanborn, Natalia P de la Ossa, Mònica Millàn, Isaac N Ruiz, Ossama Y Mansour, Mohammed Megahed, Cristina Tiu, Elena O Terecoasa, Răzvan A Radu, Thanh N Nguyen, Gioacchino Curiale, Artem Kaliaev, Alexandra L Czap, Jacob Sebaugh, Alicia M Zha, David S Liebeskind, Santiago Ortega-Gutierrez, Mudassir Farooqui, Ameer E Hassan, Laurie Preston, Mary S Patterson, Saif Bushnaq, Osama Zaidat and Tudor G Jovin in International Journal of Stroke

sj-pdf-3-wso-10.1177_1747493020959216 - Supplemental material for Cerebrovascular events and outcomes in hospitalized patients with COVID-19: The SVIN COVID-19 Multinational Registry Click here for additional data file. Supplemental material, sj-pdf-3-wso-10.1177_1747493020959216 for Cerebrovascular events and outcomes in hospitalized patients with COVID-19: The SVIN COVID-19 Multinational Registry by James E Siegler, Pere Cardona, Juan F Arenillas, Blanca Talavera, Ana N Guillen, Alba Chavarría-Miranda, Mercedes de Lera, Priyank Khandelwal, Ivo Bach, Pratit Patel, Amit Singla, Manuel Requena, Marc Ribo, Dinesh V Jillella, Srikant Rangaraju, Raul G Nogueira, Diogo C Haussen, Alejandro R Vazquez, Xabier Urra, Ángel Chamorro, Luis S Román, Jesse M Thon, Ryna Then, Emma Sanborn, Natalia P de la Ossa, Mònica Millàn, Isaac N Ruiz, Ossama Y Mansour, Mohammed Megahed, Cristina Tiu, Elena O Terecoasa, Răzvan A Radu, Thanh N Nguyen, Gioacchino Curiale, Artem Kaliaev, Alexandra L Czap, Jacob Sebaugh, Alicia M Zha, David S Liebeskind, Santiago Ortega-Gutierrez, Mudassir Farooqui, Ameer E Hassan, Laurie Preston, Mary S Patterson, Saif Bushnaq, Osama Zaidat and Tudor G Jovin in International Journal of Stroke
