# Supplementary figures and images for: Cerebrovascular events and outcomes in hospitalized patients with COVID-19: The SVIN COVID-19 Multinational Registry
Source: Int J Stroke. 2020 Sep 30;16(4):437–47. doi: 10.1177/1747493020959216 (PMC7533468; doi:10.1177/1747493020959216)

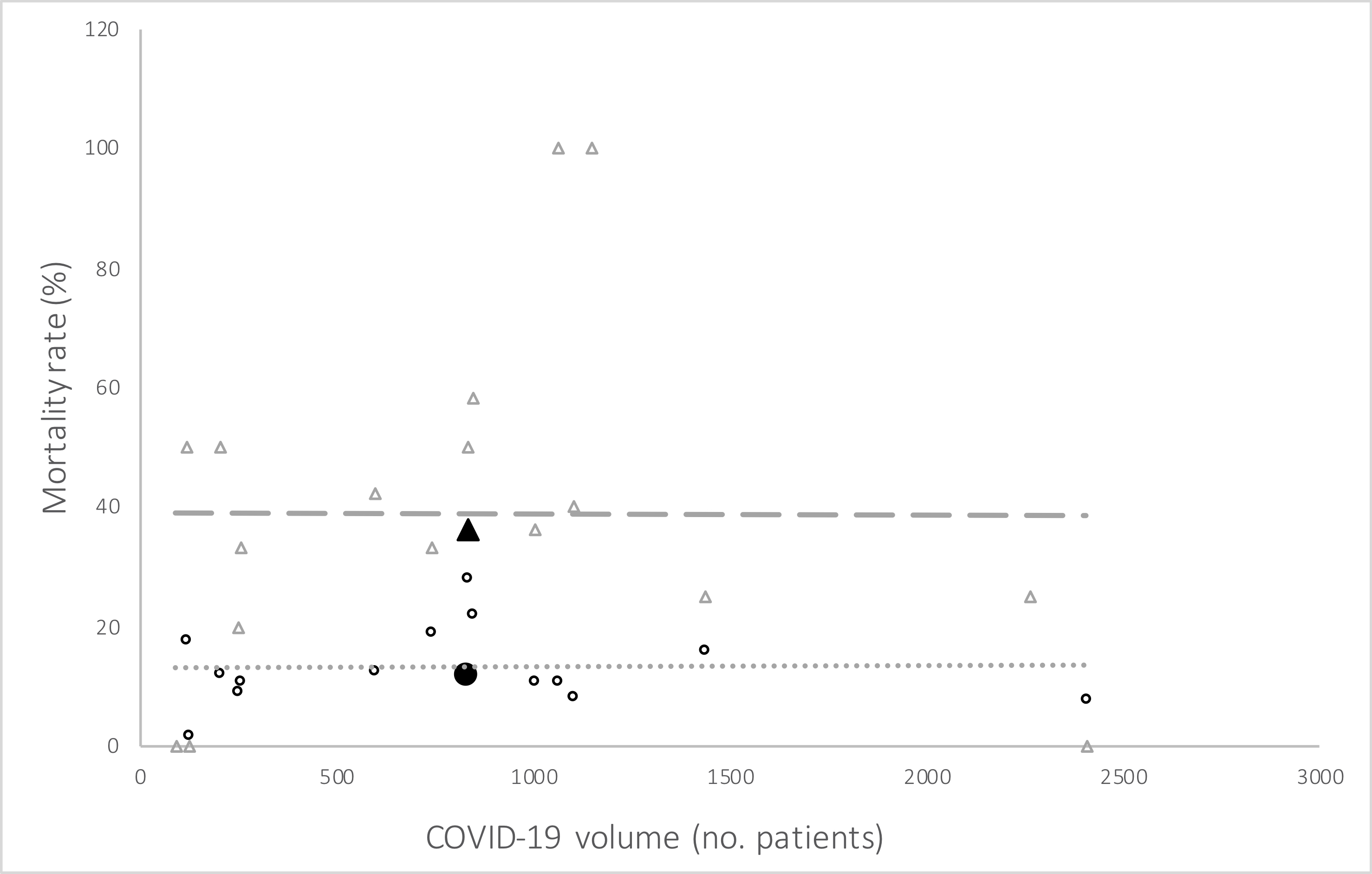

Supplement: sj-jpg-1-wso-10.1177_1747493020959216 - Supplemental material for Cerebrovascular events and outcomes in hospitalized patients with COVID-19: The SVIN COVID-19 Multinational Registry [file sj-jpg-1-wso-10.1177_1747493020959216.jpg]

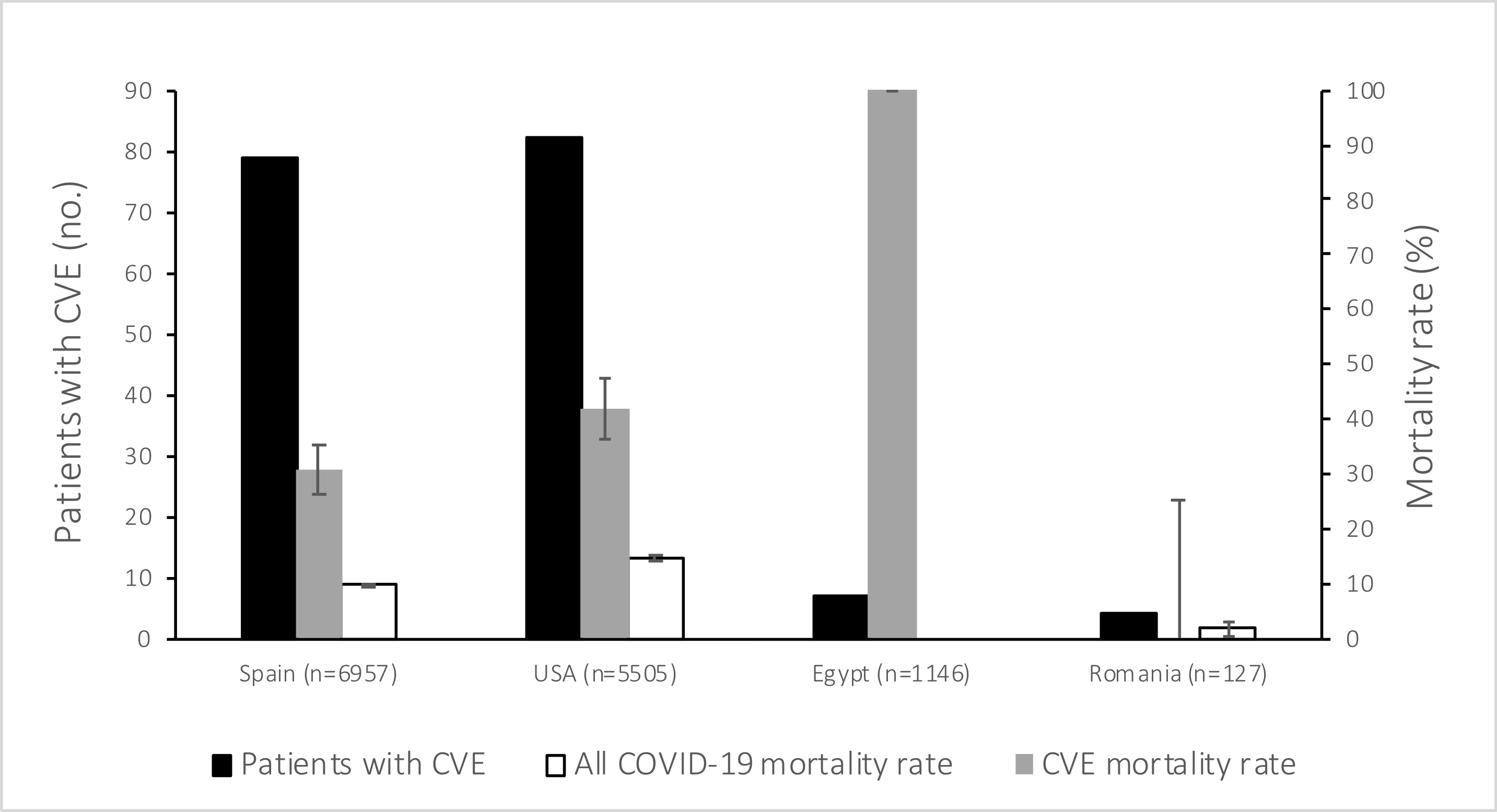

Supplement: sj-jpg-2-wso-10.1177_1747493020959216 - Supplemental material for Cerebrovascular events and outcomes in hospitalized patients with COVID-19: The SVIN COVID-19 Multinational Registry [file sj-jpg-2-wso-10.1177_1747493020959216.jpg]
